# Syngamoniasis in Tourist

**DOI:** 10.3201/eid1112.050713

**Published:** 2005-12

**Authors:** Jose C. da Costa, M.L. Delgado, Paulo Vieira, Abel Afonso, Bebiana Conde, John H. Cross

**Affiliations:** * National Institute of Health, Porto, Portugal; † Hospital Center Vila Real-Peso da Regua, Porto, Portugal; ‡ Uniformed Services University of the Health Sciences, Bethesda, Maryland, USA

**Keywords:** Brazil, Portugal, gapeworm, syngamus, Mammonogamus, letter

**To the Editor:** *Mammonogamus laryngeus* (*Syngamus laryngeus*) is a nematode parasite found in the larynx of tropical mammals (*1*), especially cattle and cats and occasionally humans (*2*). We report a case in a 65-year-old Caucasian man who visited Brazil from July 20 to September 9, 2004. The patient stayed in Rio de Janeiro and Ilhéus in northern Brazil. He ate local food, including salads, raw vegetables and fruits, and drank what he assumed was safe water.

Upon return to Portugal in September 2004, the patient experienced a cough and fever. He was seen in an emergency service and chest radiograph indicated infiltration in the left inferior lobe, the right basal hilum, and right apex. A complete blood count revealed a leukocyte count of 9,700/mm^3^, 81% polymorphonuclear leukocytes and 2.1% eosinophils. He was treated with antimicrobial drugs; a week later a radiograph showed bronchovascular markings. The patient failed to follow recommendations and in mid-October, he returned to the hospital with a persistent cough and expectoration.

In late November the patient had a persistent cough with hemoptysis. He was given antimicrobial drugs; a computed tomographic scan showed an infiltration, a sequela to pneumonia, localized in the left superior lobe. Symptoms persisted, and bronchofibroscopic examination in January 2005 showed thickening of the bilateral bronchovascular bundles and discrete diffuse inflammation in the bronchial mucosa. A Y-shaped worm, moving and wrapped in viscous, bloody mucus, was seen around the right medial bronchus. A worm was seen in the left main bronchus and, upon closer examination, a male and female worm in copula were seen. The worms removed with forceps and identified as *M. laryngeus* (Figure). Eggs from the female were characteristic of the species.

**Figure:**
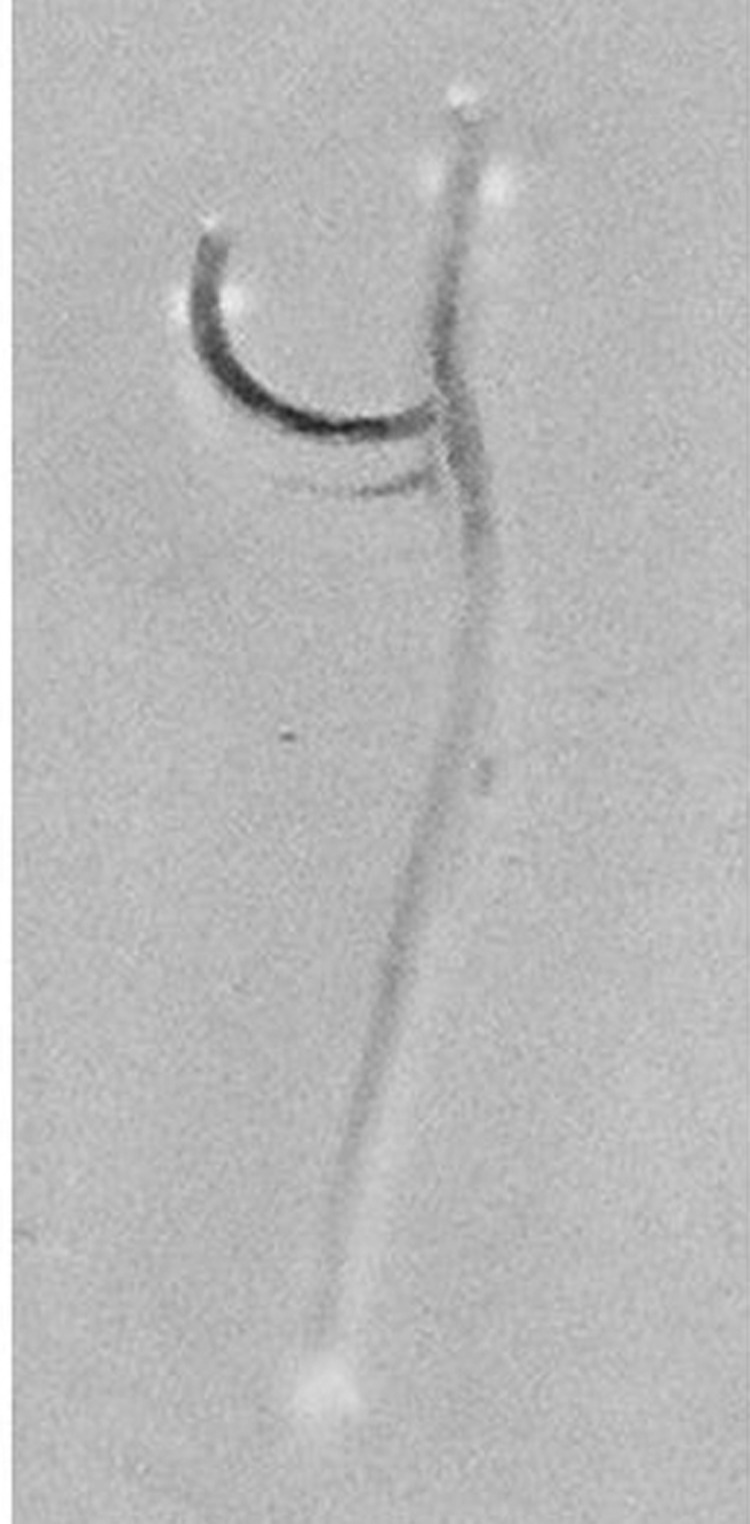
Male and female *Mammonogamus laryngeus* recovered from the bronchial mucosa.

The patient was treated with albendazole 200 mg, 3×/day for 3 days, followed by mebendazole 100 mg, 3×/day for 3 days. The cough and hemoptysis clinically improved and abated by early February.

The genus *Mammomonogamus* consists of 2 major species, *M. laryngeus* and *M. nasicola*. The former is a parasite of the laryngotracheal region of bovids and felines, and the latter is found in the nasal fossa of bovids. *M. laryngeus* and *M. nasicola* belong to the family *Syngamidae* that contains the gapeworm of birds, *S. trachea*.

Possibly 100 human infections (*3*), most caused by *M. larygeus*, have been reported from the Caribbean Islands and South America, especially Brazil, with other reports from Australia, Canada, the United States, France, United Kingdom (*4*), the Philippines (*2*), Thailand (*5*), and Korea (*6*). Many of the cases reported outside of the Caribbean and South America were usually acquired while the patient was visiting areas where *M. larygeus* was endemic. Naturally infected ruminant host are found in tropical America, India, Africa, Malaysia, the Philippines, and Vietnam (*7*).

*M. laryngeus* is blood red; the males are joined permanently to the female and are characteristically Y shaped (Figure). The males are ≈3 mm and the females are ≈10 mm in length. The mouth opening is wide, and the buccal capsule is cup-shaped with 8–10 small teeth. The worms attach to the mucosa of the larynx in animals and cause bronchitis and cough.

The means of transmission of *M. laryngeus* is unknown but it is assumed to be similar to that of *S. trachea*, which is acquired by ingesting an embryonated egg, hatched larvae, or a paratenic host such as earthworms, snails, or arthropods. The patient in our case could have been infected by eating contaminated raw vegetation or drinking contaminated water while traveling through Brazil. The life cycle of *M. laryngeus* is not completely known, but it is assumed to be similar to *S. trachea*, which penetrates the intestinal wall and migrates through the body of the animal to the tracheolaryngeal region (*8*). Eggs produced are deposited in the tracheal mucosa, swallowed, and pass in the feces.

Chronic cough and fever are the major symptoms associated with *M. laryngeus* in humans, with occasional reports of hemoptysis when the worms are in the bronchus. Worms in the larynx may cause irritation and a crawling or scratching sensation. Symptoms of asthma have been reported, and leukocytosis and eosinophilia may occur. Our patient had respiratory symptoms, persistent cough, and hemoptysis, without leukocytosis or eosinophilia.

The diagnosis of parasitosis is usually made by finding expectorated worms or visualizing by bronchoscopy and removal by forceps. Eggs may be found in sputum or feces. In our case, eggs were not found in sputum or feces.

The worms are coughed up by the patient or removed with forceps during bronchoscopy. When antihelmintics such as mebendazole and albendazole have been used, patients have reported improvement.

Although mammomonogamiasis may not be considered an emerging parasitosis, physicians should be aware of the condition especially in patients with pulmonary symptoms who visited disease-endemic areas.
